# Supplementary material for: Asymmetries of Left and Right Adrenal Glands in Neural Innervation and Glucocorticoids Production
Source: Int J Mol Sci. 2023 Dec 14;24(24):17456. doi: 10.3390/ijms242417456 (PMC10743655; doi:10.3390/ijms242417456)

Supplementary figures

Figure S1 Morphology, histology, and lipid storage between left and right adrenal glands in mice

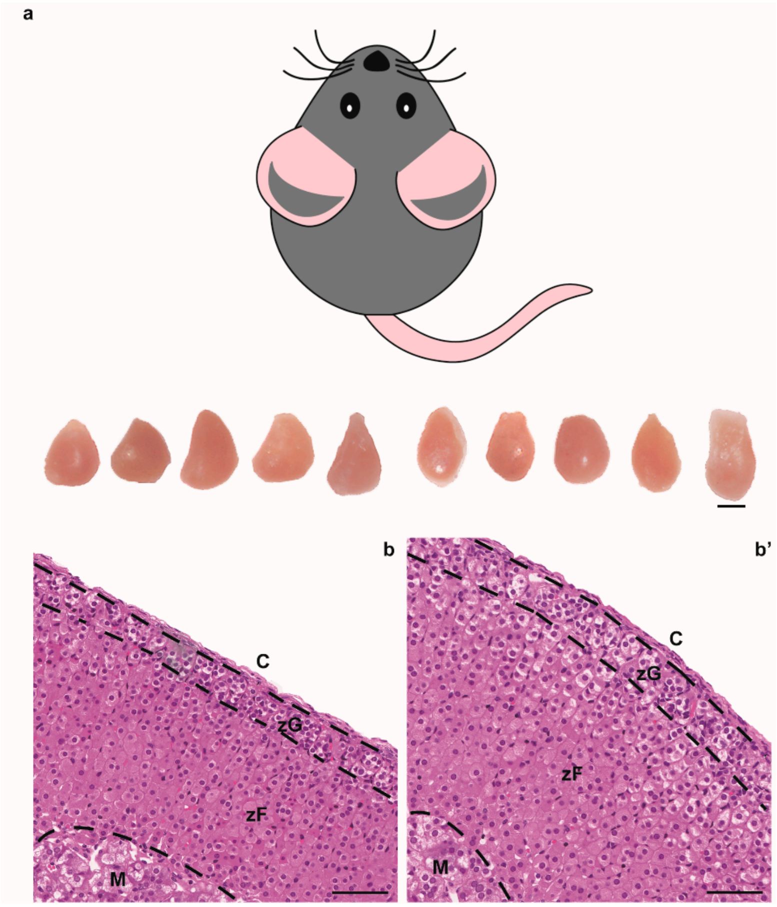

Comparison of morphology (**a**) and histology (**b**) between left and right adrenal glands. Scale bars are 0.5 mm and 100 μm respectively.

Figure S2 Hierarchical dendrogram of biological replicates between the left and right adrenal gland RNA sequencing.

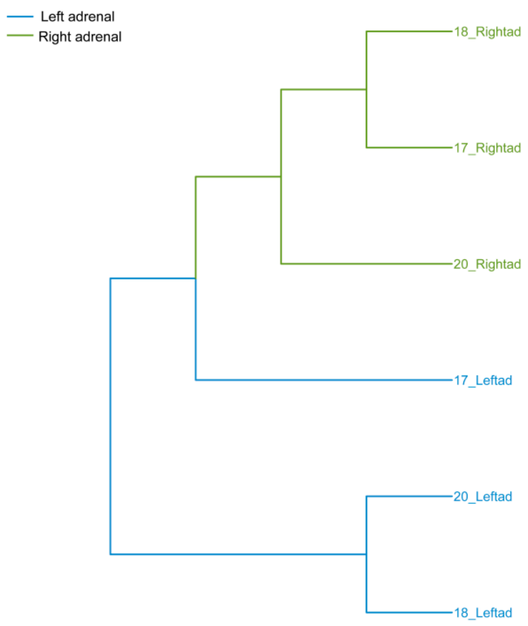

Figure S3 PCA plot of biological replicates between the left and right adrenal gland RNA sequencing.

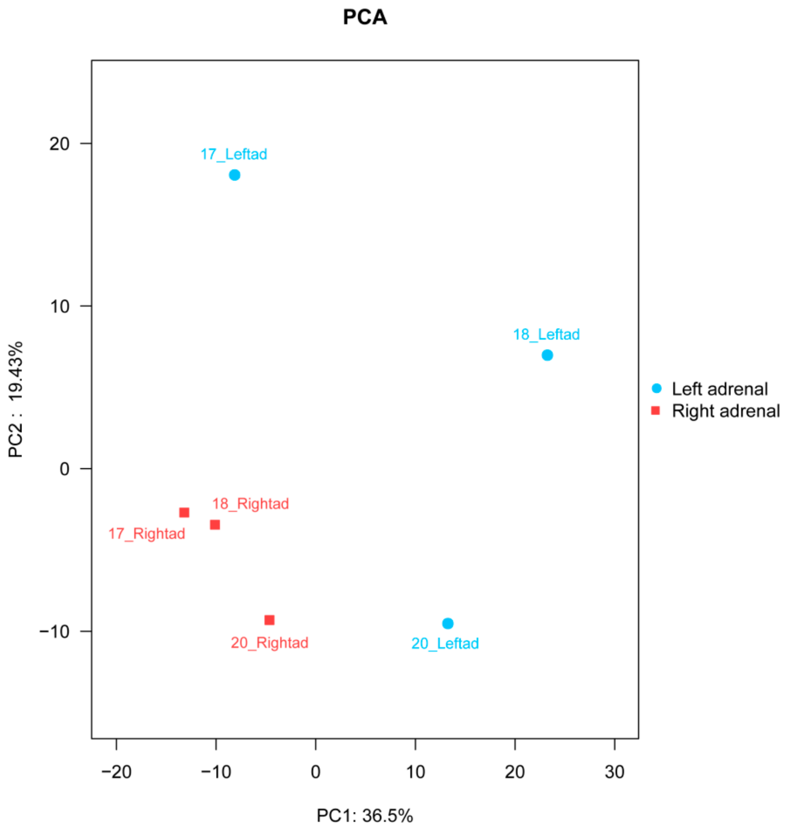

Figure S4 Top 10 significantly enriched GO terms in three GO classifications BP, CC and MF

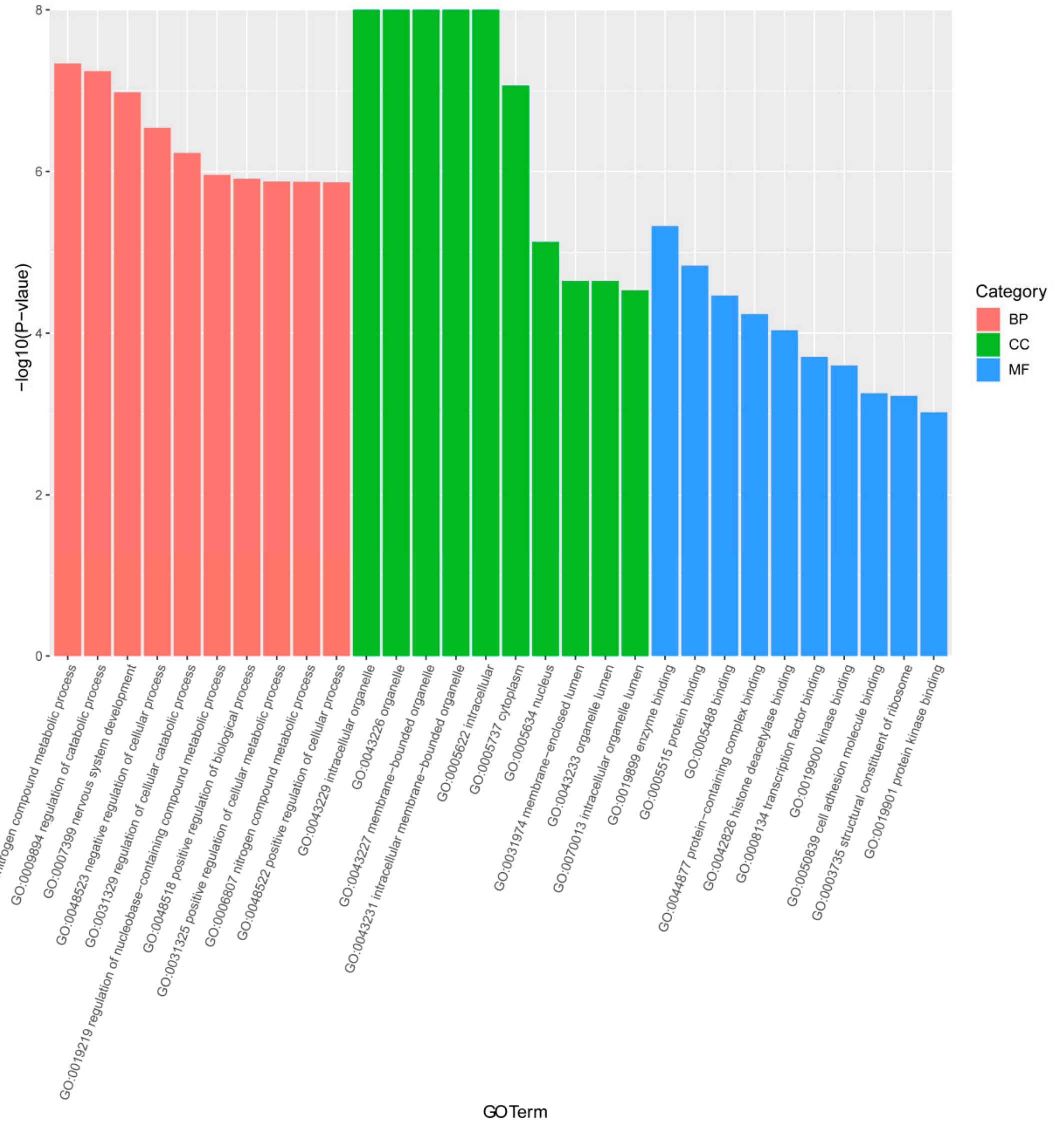

Supplement: Supplementary file 1 [file ijms-24-17456-s001.zip › Supplementary231115.pdf]
